# Development of the Antioxidant Property of Seagrass Extract-Based Hydrogel for Dental Application

**DOI:** 10.7759/cureus.54544

**Published:** 2024-02-20

**Authors:** Narayanan Sarvesh, KLG Afeeza, Vasugi Suresh, Elangovan Dilipan

**Affiliations:** 1 Physiology, Saveetha Dental College and Hospitals, Saveetha Institute of Medical and Technical Sciences, Saveetha University, Chennai, IND

**Keywords:** oral health care, antioxidant, enhalus acoroides, seagrass extract, polyacrylamide hydrogel

## Abstract

Background

Seagrass is rich in antioxidants, which can help neutralize harmful free radicals in the oral cavity. Free radicals can contribute to oxidative stress, inflammation, and various oral health issues. Incorporating seagrass extract into a hydrogel can enhance its antioxidant capacity, providing a protective effect for oral tissues. The hydrogel, composed of a biocompatible base, ensures that the material is well-tolerated by oral tissue. This is crucial for any dental application to avoid adverse reactions.

Aim

This work aimed to develop an antioxidant hydrogel that incorporates seagrass extract, with a specific emphasis on its possible use in dentistry.

Methods

A seagrass sample was collected, and its bioactive compounds were extracted through the utilization of methanol, and subsequent filtration was done. The resulting seagrass filtrate was then integrated into a hydrogel, which was synthesized using polyacrylamide and sodium alginate. Antioxidant hydrogel underwent testing for antioxidant activity through both the 2,2-diphenyl-1-picrylhydrazyl assay and the 2,2′-azino-bis-(3-ethylbenzothiazoline-6-sulfonic acid) assay. Besides, the hydrogel functional groups were investigated using Fourier transform infrared spectroscopy, while its crystalline structure was examined using X-ray diffraction analysis.

Conclusion

Seagrass extract provides inherent antioxidant properties, and incorporating this bioactive extract into the hydrogel imparts antioxidant features. The hydrogel's controlled-release property ensures both safety and efficiency. Antioxidant hydrogel for dental applications holds the potential to improve oral health.

## Introduction

In recent years, the exploration of natural sources for biomedical applications has gained considerable momentum, driven by a growing awareness of the potential drawbacks associated with synthetic materials. Antioxidants are essential in biological systems since they serve many activities, such as safeguarding against oxidative harm and participating in cell signaling pathways. Their main goal is to lessen the harm that reactive oxygen species (ROS), like superoxide radicals, hydroxyl peroxide, and nitric acid radicals, cause. ROS are produced during excessive metabolism in living organisms and can lead to extensive oxidative damage, contributing to age-related degenerative diseases, cancer, and various other human health issues. Submerged marine angiosperms known as seagrasses effectively thrive in tidal and subtidal zones throughout all oceans, excluding the polar [1]. Seagrass thrives and generates a significant number of organic materials, producing many structurally different secondary metabolites [2]. Seagrass has antioxidant and pharmacological properties owing to the presence of strong bioactive metabolites [3]. Flavonoids, phenols, citric acid, ascorbic acid, polyphenolic chemicals, terpenes, alkaloids, and reductases not only contribute to the structural complexity of seagrasses but also serve a pivotal role as formidable reducing agents [4]. The scavenging and antioxidant properties of flavonoids are influenced by the positioning of substituents. Flavonols with ortho or para hydroxyl groups within the two phenyl rings exhibit robust antioxidant characteristics, while a free hydroxyl at the 5,7-position has been demonstrated to have a pro-oxidant effect [5].

Several synthetic antioxidants, such as butylated hydroxyanisole, butylated hydroxytoluene, and tert-butylhydroquinone, are readily available on the market and are now in use. Nevertheless, their utilization is now limited due to associated side effects that may cause stomach cancer at high doses [6]. In response to concern surrounding synthetic antioxidants, there’s rising interest in discovering new alternatives. Hydrogels possessing water contents ranging from 56% to 58% exhibit mechanical characteristics akin to tendons and ligaments [7]. The increasing interest in robust and resilient hydrogels in recent years stems from their potential application in various load-bearing sectors, notably in biomedical and medical applications [8]. Recent studies have emphasized that extracts from various plants demonstrate antioxidant activity. Additionally, specific molecules that have been isolated from plant sources are being recognized for their potential as antioxidants. Moreover, some studies have indicated a connection between the antioxidant properties of these compounds and their ability to hinder cell proliferation [9]. Seagrasses are abundant reservoirs of secondary metabolites, with particular emphasis on simple, conjugated, and polymeric phenol compounds.

Seagrasses have been identified as an abundant reservoir of antioxidant compounds, a characteristic that has been leveraged in traditional folk medicine for various therapeutic applications. Notably, phenolic compounds stand out as particularly potent antioxidants, renowned for their exceptional ability to neutralize harmful free radicals and reactive species [6]. Hydrogel is a three-dimensional structure composed of hydrophilic polymers characterized by a substantial ability to engage with and retain significant quantities of water and biological fluids. This capability arises from various functional groups within the polymer chain [10]. Upon interaction with biological tissue, the swelling characteristic of the hydrogel blurs the demarcation between the hydrogel and the tissue. This effect diminishes surface tension and mitigates the adhesion of cells and proteins, consequently lowering the foreign body reaction. Additionally, the absorption of water by hydrogels results in a reduction of friction and mechanical damage to surrounding tissue [11].

Oral health plays a crucial role in influencing overall physical well-being and quality of life. The prevalence of oral diseases is a growing concern globally, affecting both developed and developing nations. Typically, oral conditions encompass issues such as dental caries, pulp necrosis, periodontitis, tooth mineralization, and more [12]. This research aims to develop a hydrogel with enhanced antioxidant capabilities using seagrass extract, therefore incorporating natural compounds into advanced biomaterials designed for dental applications. The main objective of this study is to contribute to the creation of cutting-edge dental materials, particularly tailored to promote oral health by reducing negative oxidative stress. Antioxidant hydrogel holds significant potential for dental application in oral health.

## Materials and methods

Collection of seagrass species

Fresh seagrass samples (*Enhalus acoroides*) were collected from the Thondi coastal area (9o44'05.99"N; 79o01'04.10"E) in Palk Bay, Tamil Nadu. Upon collection, the seagrass sample was transported to the laboratory, where it underwent a thorough cleansing process. The plants were washed with seawater to eliminate macroscopic epiphytes and extraneous matter, followed by a rinse with distilled water. Subsequently, the thoroughly cleaned specimens are subjected to shade drying for a duration of 10-16 days. During this drying process, the samples were exposed to air at a normal temperature, carefully avoiding direct sunlight to prevent abiotic stress. After drying, the seagrass sample was stored in a clean container.

Extraction of bioactive compounds from seagrasses sample

Seagrass samples were meticulously prepared by grinding them into a powdered form. Subsequently, a Soxhlet apparatus was employed in the extraction, utilizing 10 g of the powdered sample with 100 mL of methanol as the solvent. The resulting extract underwent filtration through Whatman No. 1 filter paper to eliminate solid particles and impurities. This filtrate was then harnessed for further analysis in subsequent studies [13].

Preparation of hydrogel

The synthesis of polyacrylamide-sodium alginate (PAM-SA) hydrogels was carried out through a meticulous procedure involving free radical polymerization. Utilizing the redox-initiating pair of ammonium persulfate (APS)/N,N,N',N'-tetramethylethylenediamine (TEMED), along with N,N-methylenebisacrylamide as a cross-linker, a standardized procedure was employed in the synthesis. The PAM-SA hydrogels were created by blending acrylamide with different quantities of sodium alginate in a water medium. The subsequent polymerization was initiated using a combination of APS and TEMED as the redox-initiating pair. After polymerization, the hydrogel was washed using distilled water to remove residual impurities. Then the hydrogel was carefully dried in an oven at 60°C to remove excess moisture content, and the obtained hydrogel was used for further studies.

Antioxidant

2,2-Diphenyl-1-Picrylhydrazyl (DPPH) Assay

The hydrogel's radical-scavenging ability was measured using the DPPH test [13]. Initially, the hydrogel was dissolved in dimethyl sulfoxide, and 0.1 mL of sample was added to 2.9 mL of a 60 µM DPPH solution. The reaction mixture was kept at 37°C in the dark for 30 minutes. After incubation, the DPPH solution's absorbance and optical density were measured at 517 nm. Use vitamin C as a positive control and compute the percentage of scavenging activity inhibition using the formula:

DPPH scavenging effect (%) = [(A0-A1)/A0] x 1000

where A0 is the absorbance of the control and A1 is the absorbance of the sample [13].

2,2′-Azino-Bis-(3-Ethylbenzothiazoline-6-Sulfonic Acid) (ABATS) Assay

Seagrass samples were tested for free radical scavenging using the ABTS radical cation decolorization assay. After mixing 7 mM ABTS in water with 2.45 mM potassium persulfate (1:1), the ABTS cation radical was kept in the dark at room temperature for 12-16 hours before use. After diluting the ABTS and solution with methanol, the absorbance at 734 nm was 0.700. After combining 5 μL of seagrass extract with 3.995 ml of diluted ABTS+ solution, absorbance was measured 30 minutes later. Each test has a solvent blank. The formula calculated a 734 nm absorbance inhibition percentage:

ABTS Scavenging effect (%) = [(Ab-Aa)/Ab) x 100]

where Ab is the absorbance of ABTS radical + methanol and Aa is the absorbance of ABTS radical + sample extract/standard. Trolox was used as a standard substance [14].

Fourier-transform infrared spectroscopy (FTIR)

Infrared spectra were acquired in the range of 4000 to 500 cm⁻¹ using an FTIR instrument in order to identify the functional groups that were present in the sample. The sample was subjected to a total of 32 scans, each of which had a resolution that was comparable to 4 cm⁻1. This was done in order to get precise and comprehensive information on the sample's composition.

X-ray diffraction (XRD)

XRD may provide insights into several aspects of a crystal's structure, including the dimensions of its lattice, the precise locations of atoms within the lattice, the identification of different phases, the degree of crystallinity, and other relevant structural features. XRD is very helpful in the fields of materials science, chemistry, and biology for the analysis of a diverse array of materials, such as minerals, polymers, and medicines. In this study, the lyophilized hydrogel material was loaded in the XRD machine, which showed the formation of the stereo complex was investigated using an X-ray diffractometer (Bruker D8 Advance) operating at a voltage of 40 kV and a current of 40 mA, employing Cu K alpha radiation (ɵ=0.15418 nm). The gel was thoroughly dried and evenly positioned on a glass slide. X-ray data were captured by scanning within an angle range of 10° to 50° at a measuring speed of 3° min⁻¹.

## Results

FTIR

**Figure 1:**
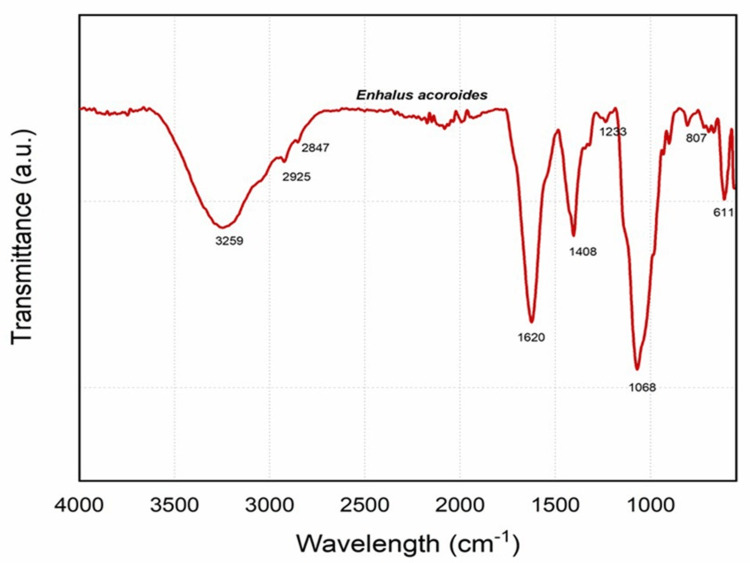
Identification of functional groups in hydrogel composed with seagrass extract using FTIR FTIR: Fourier-transform infrared spectroscopy

The FTIR spectrum of the hydrogel, made by combining polyacrylamide with *Enhalus acoroides* extract, was analyzed to determine the existence of certain functional groups in the molecule. This was done by measuring the absorption of infrared light at various wavelengths, which corresponds to the vibration of chemical bonds (Figure 1). The prominent peak at 3259 cm⁻¹ may be ascribed to the stretching vibrations of O-H bonds, which are characteristic of hydroxyl groups. This phenomenon may be attributed to the moisture content present in the hydrogel or the hydroxyl groups present in the *Enhalus acoroides* extract. The peaks seen at 2925 cm⁻¹ and 2847 cm⁻¹ are indicative of C-H stretching vibrations, which indicate the existence of methylene (-CH₂-) groups. This aligns with the polyacrylamide main structure and may include some hydrocarbon chains derived from the *Enhalus acoroides* extract. The peak seen at 1620 cm⁻¹ is likely attributed to the N-H bending of primary amines or the C=O stretching of amide groups. It is probable that this peak originates from the carbonyl stretch of the amide group. The 1408 cm⁻¹ peak is likely attributed to C-N stretching vibrations, which align with the presence of amide bonds in polyacrylamide. The peak seen at 1233 cm⁻¹ suggests the presence of C-N stretching vibrations in amine-related functional groups or maybe C-O stretching in ethers or esters, which might be part of the components found in the *Enhalus acoroides* extract. The observed absorption peak at 1068 cm⁻¹ is likely attributed to the stretching vibrations of C-O-C bonds, maybe originating from ethers or alcohols present in the polymeric network of the hydrogel. The peaks seen at 807 cm⁻¹ and 611 cm⁻¹ are likely attributed to the out-of-plane bending vibrations of =C-H groups or maybe the stretching of C-S bonds, indicating the existence of thiol groups. The inclusion of the *Enhalus acoroides* extract in the hydrogel may be expected to result in the emergence of new peaks and a modification of peak intensity in comparison to a spectrum obtained from pure polyacrylamide. The presence of particular functional groups in organic molecules, such as polysaccharides or phenolic compounds, might result in the appearance of additional peaks while extracting components.

XRD

**Figure 2:**
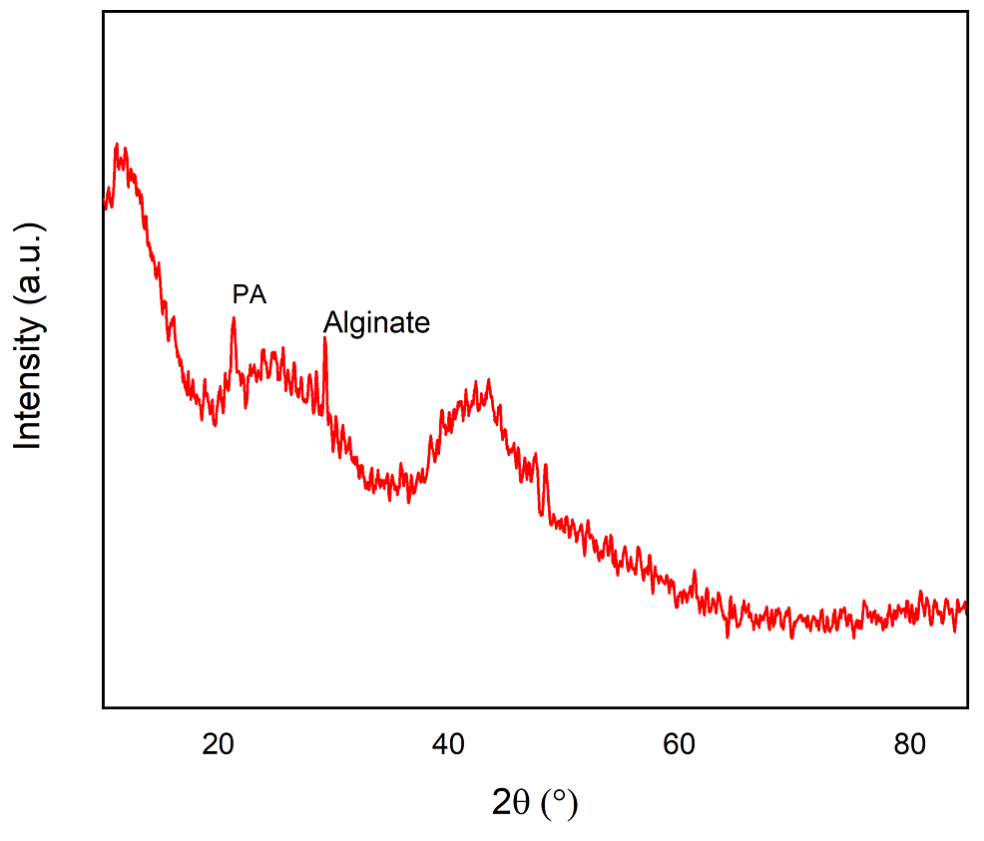
XRD pattern of polyacrylamide composed with alginate-based hydrogel film XRD: X-ray diffraction

The XRD spectrum of a hydrogel made of sodium alginate/polyacrylamide infused with *Enhalus acoroides* extract shows a combination of crystalline and amorphous properties (Figure 2). The degree of crystallinity is determined to be 29.3%, while the remaining 70.3% is composed of amorphous material. This indicates that the hydrogel has a substantial amorphous character, perhaps attributed to the disorganized polymeric structure, with a noticeable level of crystallinity. The crystallinity may result from distinct intermolecular interactions among polymer chains or the existence of crystalline domains within the infused extract. The spectrum exhibits many distinct peaks, perhaps indicating the presence of different crystalline phases inside the hydrogel. The peaks seen at an angle of 21.282° might perhaps be linked to the crystalline regions present in either the sodium alginate or the polyacrylamide. The presence of a peak at an angle of 23.677° suggests the existence of an additional crystalline phase. In a composite material, this phenomenon might arise due to the intricate interplay between the constituent elements or the existence of distinct crystalline structures resulting from the infused extract.

DPPH assay

**Figure 3:**
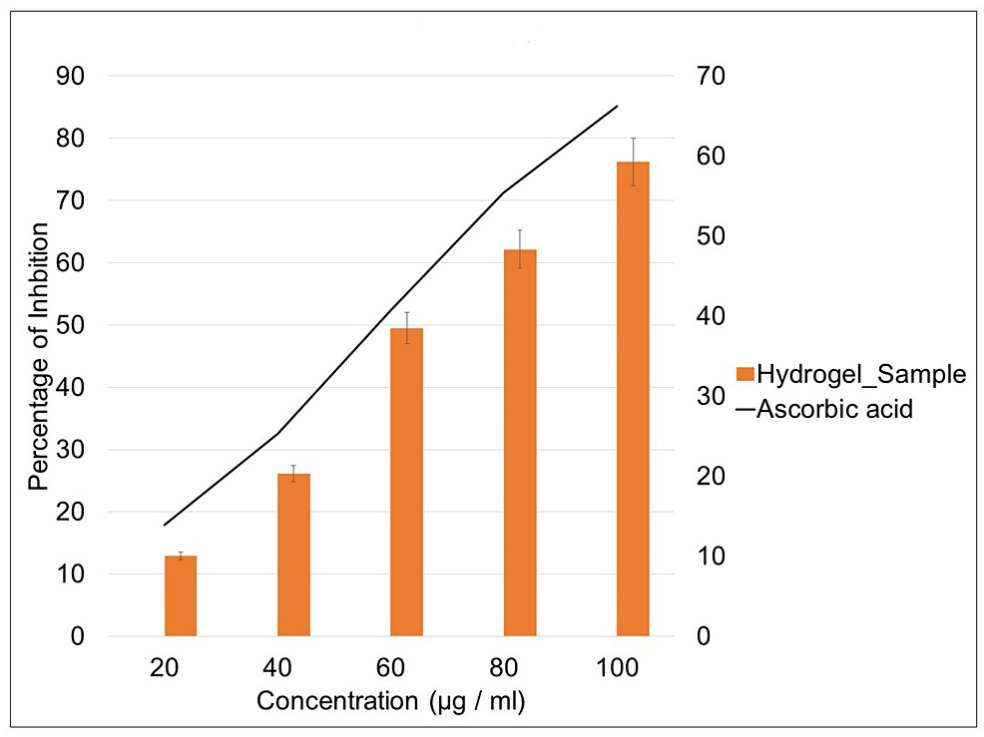
DPPH assay of hydrogel doped with seagrass Enhalus acoroides extract DPPH: 2,2-diphenyl-1-picrylhydrazyl

The investigation into the antioxidant properties of the hydrogel revealed a positive correlation between its concentration and the percentage of inhibition in the DPPH assay (Figure 3). As the concentration of hydrogel increased, there was a corresponding elevation in the inhibition percentage. Notably, the highest level of inhibition was observed at the concentration of 100 µg/ml, mirroring the pattern observed in the ascorbic acid control group. These findings suggest a dose-dependent relationship between the concentration of hydrogels and their respective antioxidant activities. The parallel outcome in the hydrogel further underscores the efficiency of the hydrogel as an antioxidant agent, comparable to the well-established antioxidant agent ascorbic acid.

ABTS assay

**Figure 4:**
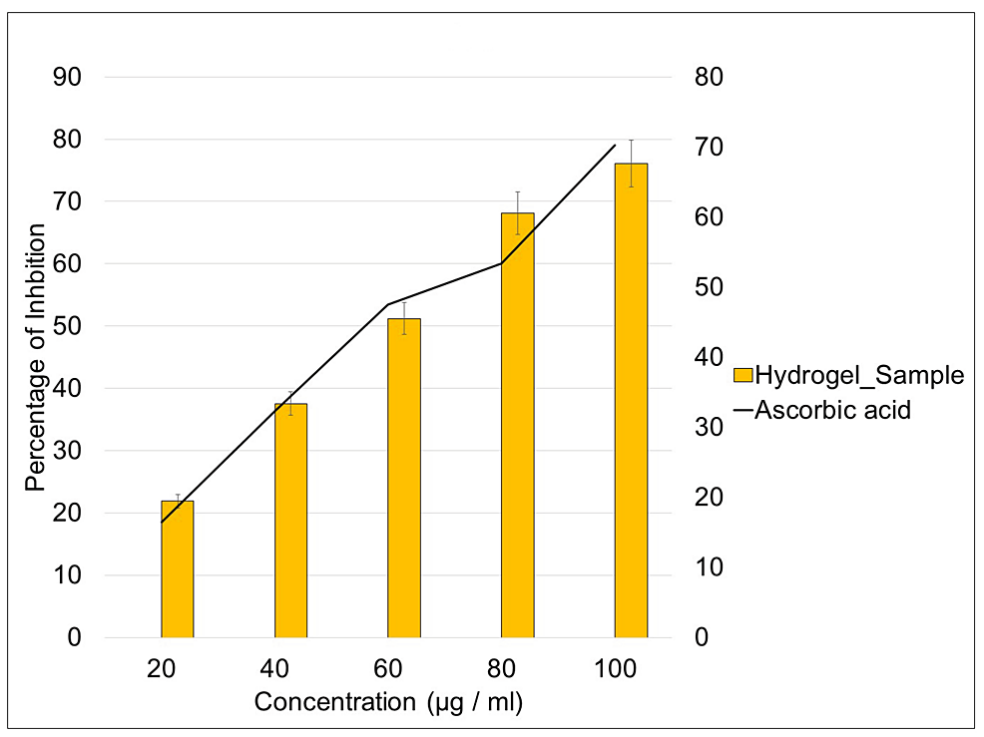
ABTS assay of hydrogel doped with seagrass Enhalus acoroides extract ABTS: 2,2′-azino-bis-(3-ethylbenzothiazoline-6-sulfonic acid)

Investigation of the antioxidant activity using the ABTS assay demonstrates a positive correlation between the concentration of the hydrogel and the percentage of inhibition (Figure 4). As the concentration increased, there was a concurrent rise in the inhibition percentage. Notably, at the concentration of 80 µg/ml, the hydrogel exhibited a higher percentage of inhibition compared to the control. Reinforcing the efficiency of the hydrogel in scavenging free radicles in comparison to the control (ascorbic acid). Overall, the highest percentage of inhibition was observed at a concentration of 100 µg/ml.

## Discussion

The development of an antioxidant property in a seagrass extract-based hydrogel for dental applications represents an innovative approach to improving oral healthcare. Seagrass, a naturally occurring and sustainable resource, offers the potential to harness bioactive compounds with antioxidant properties. Hydrogel serves as an effective carrier for antibacterial agents, facilitating their targeted delivery into periodontal pockets through injection. This delivery method proves successful in alleviating inflammation by eradicating anaerobic bacteria and impeding bacterial growth. Leveraging the adhesive and transport properties of hydrogels, medications can gradually diffuse into the periodontal pocket, ensuring prolonged and localized therapeutic effects without undesirable side effects. Hydrogel has demonstrated its suitability as a scaffold material for periodontal regenerative medicines [15]. The oral cavity is continuously exposed to diverse sources of oxidative stress, ranging from microbial infection and inflammation to dietary factors. This oxidative stress can result in detrimental effects on oral tissue, contributing to the development of conditions such as periodontal disease, dental caries, and mucosal disorders. A critical aspect of defense against these adverse effects involves the role of antioxidants. The overall redox state within the oral environment is influenced by the balance between antioxidants (such as glutathione, reduced glutathione, oxidized glutathione, glutathione peroxidase activity, superoxide dismutase, and catalase) and pro-oxidants (including reactive species and levels of nitrates and nitrites) [16]. Seagrass extract is known for its demonstrated antioxidant activity in various species, such as *Halophila ovalis*, *Posidonia (L)*, *Syringodium isoetifolium*, *Halodule uninervis*, and *C. rotundata*, which hold significant promise for application in dental care [17,18].

The research done by Tangon et al. showed that the extract of *Enhalus acoroides* had noteworthy antioxidant activity, which was supported by the existence of phenolic and flavonoid components in the seagrass. The research revealed a strong association between the overall phenol content and total flavonoid content in *Enhalus acoroides*, which is recognized for its powerful antioxidant characteristics. This correlation emphasizes the crucial function of these bioactive components in antioxidant activity [19]. Several studies have affirmed the antioxidant potential of these seagrasses. Incorporating seagrass extract into a hydrogel formulation presents a compelling avenue to harness its antioxidant properties for dental application. The synergistic effect of seagrass antioxidants could potentially contribute to mitigating oxidative stress within the oral cavity, offering a natural and sustainable approach to enhancing oral health. The evaluation of the antioxidant activity in the hydrogel-containing seagrass extract was performed using the DPPH and ABTS tests. These commonly used tests are good markers of the ability of antioxidants to scavenge radicals.

The examination of the hydrogel's antioxidant characteristics revealed significant findings, indicating a strong positive association between the hydrogel concentration and the percentage of inhibition in the DPPH experiment. The DPPH free radical is often used to evaluate the ability of a material to act as a scavenger of free radicals and a supplier of hydrogen [20]. The DPPH test relies on the neutralization of DPPH, which is a stabilized free radical. The substance is a dark-hued crystalline complex composed of stable free-radical particles. It is generally acknowledged as a well-known radical and is often used in antioxidant testing [21]. The DPPH radical, when reduced and transformed into DPPH, causes the solution to acquire a deep purple hue. However, after undergoing reduction and conversion into DPPH, it loses its color and appears colorless or pale [22,23]. Our results show a dose-dependent relationship between the concentration of the hydrogel and its antioxidant efficiency. As the concentration of the hydrogel increased, there was a corresponding elevation in the inhibition percentage. Notably, the highest level of inhibition was observed at the concentration of 100 µl/ml, indicating the optimal concentration for eliciting the maximum antioxidant effect. This concentration-dependent pattern closely mirrors the observed outcomes in the ascorbic acid control group, a well-established antioxidant agent, further supporting the robust antioxidant activity of the hydrogel.

ABTS is used as a chemical reagent to investigate the reaction kinetics of certain enzymes [24]. The transformation of ABTS into its radical cation takes place via the incorporation of sodium persulfate. The resultant radical cation has a blue hue and absorbs light with a wavelength of 734 nm. The ABTS radical cation exhibits reactivity toward various antioxidants, such as phenolics, thiols, and vitamin C [19]. The investigation into the antioxidant activity using the ABTS assay revealed a compelling positive correlation between the concentration of the hydrogel incorporated with seagrass extract and the percentage of inhibition. The observed increase in inhibition percentage with rising concentration signifies a dose-dependent response, indicating the enhanced radical-scavenging capacity of the hydrogel. Notably, at a concentration of 80 µl/ml, the hydrogel exhibited a significantly higher percentage of inhibition compared to the control (ascorbic acid), emphasizing. Its efficiency in scavenging free radicals. This outcome underscores the potential of seagrass-derived antioxidants to augment the antioxidant profile of the hydrogel. The pinnacle of the observed inhibitory effect was reached at a concentration of 100 µl/ml, suggesting an optimal concentration for maximizing antioxidant activity.

FTIR analysis offers insight into both the molecular structure and composition of a film. Additionally, it serves as a valuable tool for assessing the extent of cross-linking within the film, a factor that significantly influences its mechanical strength and durability properties [25]. The FTIR analysis conducted on the hydrogel formulated by combining polyacrylamide with *Enhalus acoroides* extract unveiled crucial information about its molecular composition. The prominent peak at 3259 cm-1 attributed to O-H stretching vibrations suggests the presence of hydroxyl groups, potentially from both the hydrogel’s moisture content and the *Enhalus acoroides* extract. The peaks at 2925 cmˉ¹ and 2847 cmˉ¹ indicate C-H stretching vibration, aligning with the polyacrylamide structure and possibly incorporating a hydrocarbon chain from the extract. The peak at 1620 cmˉ¹, suggestive of N-H bending or C=O stretching, likely originates from the carbonyl stretch of the amide group in polyacrylamide. The peaks at 1408 cm-1 and 1233 cmˉ¹ imply C-N stretching vibrations, corresponding to amide bonds in polyacrylamide and potentially ethers or esters in the *Enhalus acoroides* extract. The absorption peak at 1068 cmˉ¹ hints at C-O-C stretching vibration, potentially arising from ethers or alcohols in the hydrogel’s polymeric network. Peaks at 807 cm-1 and 611 cmˉ¹ suggest out-of-plane bending vibrations of =C-H groups or C-S stretching, indicating the presence of thiol groups. The alterations in peak patterns and intensity and the potential emergence of new peaks with the inclusion of *Enhalus acoroides* extract underscore the modification of the hydrogel’s functional groups, substantiating its suitability for diverse applications, including dental formulations, where the unique molecular composition may offer additional benefits.

The XRD spectrum of the hydrogel, composed of sodium alginate and polyacrylamide infused with *Enhalus acoroides* extract, reveals a compelling blend of crystalline and amorphous characteristics. The calculated degree of crystalline at 29.3% implies a substantial amorphous nature, possibly originating from the inherent disorganization in the polymeric structure. The noticeable level of crystallinity may arise from distinct intermolecular interactions or crystalline domains within the infused extract. The spectrum exhibits multiple distinct peaks, hinting at the presence of various crystalline phases within the hydrogel. Peaks at 21.282° may be associated with crystalline regions in either sodium alginate or polyacrylamide, while the presence of a peak at 23.677 suggests an additional crystalline phase. In a composite material, such distinct peaks might result from intricate interactions between constituent elements or the presence of unique crystalline structures influenced by the infused extract. This analysis sheds light on the complex structural composition of the hydrogel, offering insight into its potential applications, especially in areas where a controlled combination of crystalline and amorphous properties is desirable, such as in dental formulations or other biomedical applications.

Limitations

The biocompatibility of the materials utilized is a major consideration in dental applications. The research should investigate the safety of using the seagrass extract-based hydrogel in the oral cavity, particularly during extended periods of exposure. Extensive biocompatibility testing, both in vitro and in vivo, may be necessary. Hydrogels have the potential to experience deterioration or alterations in their physical and chemical characteristics as time progresses, which may affect their efficacy as dental materials. The research should examine the enduring stability of the hydrogel over an extended period of time, as well as its capacity to act as an antioxidant. Clinical studies are crucial for establishing the safety and effectiveness of any novel dental material or therapy. The study should recognize the need for more research, such as clinical trials, prior to the extensive use of dental applications.

## Conclusions

The development of an antioxidant-rich hydrogel derived from seagrass extract for dental applications presents a promising breakthrough in oral health care. Seagrass, which has a high amount of natural antioxidants, is becoming recognized as a sustainable and environmentally friendly remedy for combating oxidative stress and inflammation in the mouth. The successful extraction and integration of these antioxidants into the hydrogel matrix establishes a biocompatible and controlled-release platform, ensuring the safety and efficiency of the product in dental applications. Despite significant progress, further research is imperative to optimize formulations, assess stability, and explore broader applications in oral health care. A natural-inspired dental care approach holds exciting potential for enhancing oral well-being, emphasizing the transformative impact of harnessing antioxidant hydrogel in dental applications.
